# Supplementary material for: Transition from undergraduates to residents: A SWOT analysis of the expectations and concerns of Japanese medical graduates during the COVID-19 pandemic
Source: PLoS One. 2022 Mar 30;17(3):e0266284. doi: 10.1371/journal.pone.0266284 (PMC8967019; doi:10.1371/journal.pone.0266284)
Supplement: S1 Table — (DOCX) [file pone.0266284.s001.docx]

**S1 Table. Summary of themes and sub-themes from the SWOT analysis, with example quotes**

| **Strength** |
| --- |
| 1. Positive mindset    1. Resilience during the COVID-19 pandemic   *I've become more resistant to uncertain situations, or maybe I've become mentally stronger because of everything I've had to endure.*   - 1. Coping with stress   *I think my resistance to uncertain situations has strengthened, and the self-restraint developed during my undergraduate days gives me hints on how to cope with stress during my residency.*   - 1. Awareness regarding professionalism   *Since medical school, I have been highly conscious of the fact that our actions can expose others to infection.*   1. Awareness regarding self-management    1. Strict infection control and physical condition   *During conventional residency training, even if we were feeling slightly sick, it was difficult to talk about it, and we felt obliged to go to the hospital to work. Since the trend has changed, people are encouraged to take a break if they are not feeling well under the pandemic. Residents are now able to properly communicate their health conditions to others and avoid* *overworking, which builds resilience.*   - 1. Self-efficacy   *It's been a year since I've been in clinical practice and there are still many things I don't understand, but I can feel myself growing every day.* |
| **Weakness** |
| 1. Concerns pertaining to clinical competency    1. Building a good relationship with patients   *Because of the pandemic, I hadn’t been in contact with patients and SPs for over a year, so I was quite worried about communicating with patients.*   - 1. Limitations of simulation education   *When I go out into the clinical field, I realise that simulations and people are different.*   - 1. Anxiety about accurate physical examination   *I cannot deny that I have some concerns about my own physical examination skills.*   1. Unfamiliarity with the clinical environment    1. Feeling of unfamiliarity with the work environment   *I’m struggling with hand washing and gowning techniques when I enter the operating room, and I don’t know the routine work of the department that I’m rotating through, so I’m trying my best to get used to the clinical setting.*   - 1. Concerns about supervising physicians and other professionals   *All the supervisors and nurses seem to be busy during the pandemic, so I’m not sure if I can talk to them casually.* |
| **Opportunity** |
| 1. Room for growth    1. Careful communication with patients   *When I was in medical school during the pandemic, I mainly studied remotely and did not have many opportunities to interact with patients, but I feel that I have acquired the habit of studying on my own, and I am sure that this habit will be utilised during my training.*   - 1. Self-directed learning style   *After I enter the clinical field, I will try to carefully interview and examine each patient and follow up with them on a long-term basis.*   1. Cooperation with others    1. Expectation of being an immediate asset   *I want to be actively involved with COVID-19 patients so that I can hone my skills and soon be useful in the field.*   - 1. Building relationships with supervisors   *Since I did not have many opportunities to interact with others, I feel that I can create a good training environment by actively getting involved and building relationships with my supervisors.*   - 1. Community building among residents   *Since the start of training, residents have been able to talk with each other about various things. All the residents get along well, and we are connected through social networking, so it is easy for us to share information with each other under the COVID-19 pandemic.* |
| **Threat** |
| 1. Inadequate socialisation    1. Limited career choices   *Since I couldn’t rotate departments, I wanted to be in the off-campus training while in medical school … I am under a lot of pressure to choose my department in the limited time I have during my upcoming residency training period.*   - 1. Adjustment as a member of society   *The orientation was also online, so I don’t really feel like I’m out in the medical field yet; I feel like I’m extending my student days.*   1. Uncertainty during the COVID-19 pandemic    1. Stress associated with stringent infection control measures   *I’m in pain when I’ve done my PCR tests, and I’m on the ward with good infection prevention, but I think it’s a threat that I’m at risk of becoming the centre of the cluster as I haven’t taken my full dosage of the vaccination.*   - 1. Physical and mental burdens   *Since I started my training on the ward, I’ve been more nervous. I also experienced some hyperventilation, which may be an effect of the training.*   - 1. Uncertainty pertaining to the clinical training environment   *I might get infected from a patient, or there might be a possibility of residents passing it on to each other or something like that. I don't want to bother people, but...* |
