# Supplementary material for: Transition from undergraduates to residents: A SWOT analysis of the expectations and concerns of Japanese medical graduates during the COVID-19 pandemic
Source: PLoS One. 2022 Mar 30;17(3):e0266284. doi: 10.1371/journal.pone.0266284 (PMC8967019; doi:10.1371/journal.pone.0266284)
Supplement: S2 Table — (DOCX) [file pone.0266284.s002.docx]

**S2 Table. Summary of themes and sub-themes depicting desired support for transition, with example quotations**

| **Desired support for transition** |
| --- |
| 1. Specific instructional methods    1. Step-by-step guidance   *At first, if I had a chance to watch a senior doctor doing something on the side, I would be able to imitate what she was doing. I don't have any experience with gown technique, so I would like her to teach it as if she were teaching to beginner, even more so during surgical training.*   - 1. Hands-on experience under direct observation   *There are many things that I can’t do even though I’ve prepared well in my own way, especially during procedures. I would like them to keep an eye on me.*   - 1. Detailed feedback   *I’ve been in a situation where I’m not sure if I’m really doing the right thing when I go out into the clinical field, so I’d appreciate it if my supervisor could give me detailed feedback on what I’m doing ...*   1. Training support system    1. Clarification regarding training policies   *When I first started my training, I didn't know how I should train or what kind of training I should do, so I thought it would be easier to learn if the department decided on a training policy for their specialty.*   - 1. Efficient information gathering   *It would be nice if the residency manual is not only prepared but also directly tells us what is important and prioritised in that training department.*   - 1. Support by fellows   *I was very impressed that a fellow who was close to my grade taught me in detail the technique of central venipuncture using a simulator, and it was easy for me to ask* *questions.*   1. Cordial relationship    1. A sense of closeness   *I’ve been hesitant because I’m worried about the procedure, so it would be nice if the supervisors could talk to me. It was reassuring to be told that I could always ask for help if I didn’t understand something ...*   - 1. Support without discrimination   *I’m from another country, and there are many residents from different universities, so I’m really worried, but I’m happy to be involved regardless of that.* |
